# REVIEWING TRANSFERABILITY IN ECONOMIC EVALUATIONS ORIGINATING FROM EASTERN EUROPE

**DOI:** 10.1017/S0266462315000677

**Published:** 2015

**Authors:** Olena Mandrik, Saskia Knies, Zoltan Kalo, Johan L. Severens

**Affiliations:** iBMG–Institute of Health Policy & Management; Erasmus University RotterdamOlena.dem@gmail.com; iBMG–Institute of Health Policy & Management, Erasmus University Rotterdam; National Health Care Institute; Department of Health Policy and Health Economics, Eötvös Loránd University; Syreon Research Institute; iBMG–Institute of Health Policy & Management; iMTA–Institute of Medical Technology Assessment; Erasmus University Rotterdam

**Keywords:** Cost effectiveness, Review, Developing countries

## Abstract

**Objectives:** The aim of this study is to analyze the quality and transferability issues reported in published peer-reviewed English-language economic evaluations based in healthcare settings of the Central and Eastern European (CEE) and former Soviet countries.

**Methods:** A systematic search of economic evaluations of healthcare interventions was performed for Armenia, Azerbaijan, Belarus, Bulgaria, Estonia, Georgia, Turkmenistan, Kazakhstan, Lithuania, the former Yugoslav Republic of Macedonia, Republic of Moldova, Romania, the Russian Federation, Serbia, Slovenia, and Ukraine. The included studies were assessed according to their characteristics, quality (using Drummond's checklist), use of local data, and the transferability of inputs and results, if addressed.

**Results:** Most of the thirty-four economic evaluations identified were conducted from a healthcare or payer perspective (74 percent), with 47 percent of studies focusing on infectious diseases. The least frequently and transparently addressed parameters were the items’ stated perspectives, relevant costs included, accurately measured costs in appropriate units, outcomes and costs credibly valued, and uncertainties addressed. Local data were often used to assess unit costs, baseline risk, and resource usage, while jurisdiction-specific utilities were included in only one study. Only 32 percent of relevant studies discussed the limitations of using foreign data, and 36 percent of studies discussed the transferability of their own study results to other jurisdictions.

**Conclusions:** Transferability of the results is not sufficiently discussed in published economic evaluations. To simplify the transferability of studies to other jurisdictions, the following should be comprehensively addressed: uncertainty, impact of influential parameters, and data transferability. The transparency of reporting should be improved.

## INTRODUCTION

The application of health technology assessment (HTA), a policy analysis that examines short- and long-term consequences of the use of a health technology in decision making (1), has significantly increased during the past years all around the world (2;3). At the same time, middle income countries, classified by the World Bank as countries with a gross national income per capita between $1,036 US and $12,475 US (4), face common problems in establishing HTA paradigms (2). Most countries of the Central and Eastern European (CEE) and former Soviet Union regions are middle income countries, while many others from the same regions (for example, the Russian Federation), being nominally high-income markets, still possess “middle-income characteristics” (2).

Among countries of the CEE region, Poland, the Czech Republic, Slovakia, and Hungary have introduced HTA principles and so can be considered countries with an established HTA process (5;6). The other CEE and former Soviet countries, being in different stages of HTA implementation, frequently incorporate some HTA elements or emerge with an idea for HTA use in their healthcare decision making. Frequently, such countries have no well-defined structural plan for the implementation of HTA results in their healthcare decision-making process. Some of them express initiation for full or partial HTA implementation, while not being able to allocate significant financial or qualified scientific resources for substantiating policy decisions with evidence (6;7). In many countries (e.g., Hungary and Poland), HTA capacity building is a first mandatory step for HTA implementation, followed by the development and approval of methodological guidelines and, after having an appropriately organized scientific environment, use of compulsory HTA in policy decisions (7;8). In other countries (e.g., Slovakia) mandating HTA evidence before pricing and reimbursement decisions of pharmaceuticals is the first step of HTA implementation, which eventually creates the need for HTA training. However, insufficient or low-quality HTA capacity may lead to speculations and corruption rather than the benefits from early HTA implementation.

The other challenge for CEE and former Soviet countries with no central HTA agency is that when voluntary HTA dossier submissions exist, HTA may become a commercial promotional product rather than a decision-making tool. Although pharmaceutical companies, consulting firms, or private HTA agencies may become interested in this particular topic, the actual need for such an assessment is not always expressed by the government. For example, while health authorities may be equally interested in HTA for expensive medical services and procedures, most of the online Russian-language literature on HTA studies, which was acquired by means of an Internet search, is focused on pharmaceuticals.

Although HTA capacity is already considered to be very limited (2), the implementation of HTA research and the critical appraisal of completed studies in CEE and former Soviet countries with no single public HTA agency may involve several additional problems (6). When the appropriate training in HTA methodologies and concepts (and more specifically economic evaluations, being the core concept within HTA) is provided to experts from national institutions with no formal education in HTA or related sciences, there is no guarantee that the training will be successful. Language barriers limit the impact of international training courses in English. Language limitations, together with quality considerations, are factors that influence the potential transferability and generalizability of local-language studies, which are frequently not referenced in the international databases.

The potential solution while operating in a narrow pool of high-quality economic studies can be generalizability or simplified transferability of economic evaluations across countries with defined similarities in healthcare systems and economic development (6). The need for simple transferability of health economic studies is potentially more important in countries with limited scientific and financial resources for conducting economic evaluations, as is the case in many countries of the CEE and former Soviet Union (2;5;7). In this study, we analyzed the scope of transferability issues that are addressed in published peer-reviewed English-language economic evaluations based in healthcare settings of CEE and former Soviet countries with a recently formed or no centralized HTA agency. The research aim was operationalized by the following research questions: (i) What are the background characteristics of economic evaluations conducted in healthcare settings of CEE and former Soviet countries and published in English-language peer-review journals? (ii) What is the quality of the retrieved studies based on Drummond's check-list for assessing economic evaluations? (iii) To what extent is the transferability of economic evaluations addressed in the retrieved studies? (a) In what respect were local and foreign inputs used in economic evaluations? (b) Are the transferability of the inputs and the results of the study frequently discussed in these publications?

## METHODS

In September 2013, a systematic search for scientific literature on economic evaluations conducted in the selected CEE and former Soviet countries was performed (Armenia, Azerbaijan, Belarus, Bulgaria, Estonia, Georgia, Turkmenistan, Kazakhstan, Lithuania, the former Yugoslav Republic of Macedonia, Republic of Moldova, Romania, Russian Federation, Serbia, Slovenia, and Ukraine). The methodology applied in this review was based on the recommendations of the Centre for Reviews and Dissemination guidance for undertaking reviews in healthcare by the University of York (9).

### Data Search

**Figure 1.**
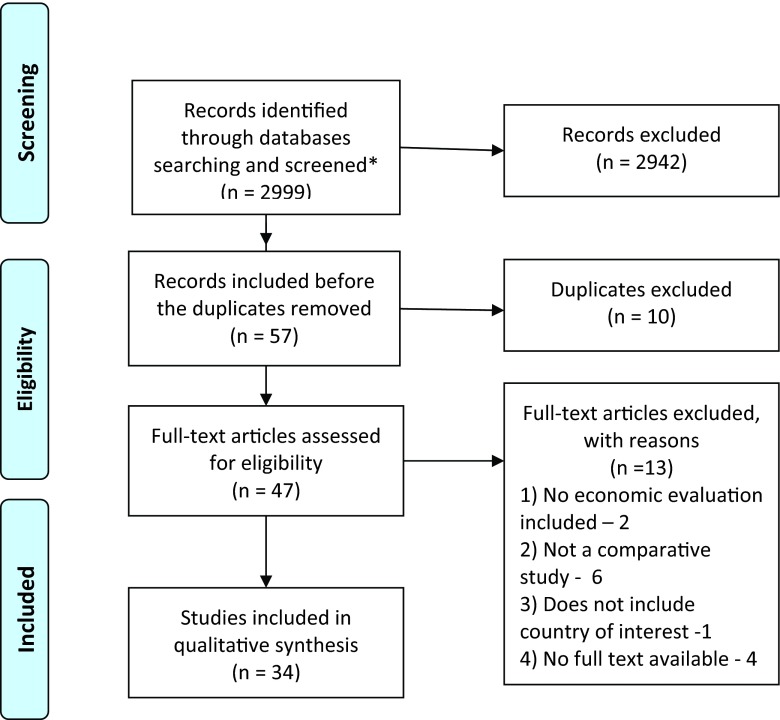
Flowchart outlining paper selection process for the systematic review.

The search, selection, and analysis of the relevant articles were performed in a three-step procedure: initial assessment of the title, abstract, and keywords (Step 1); a full-text assessment of the selected references (Step 2); and analysis of the articles that fully corresponded to the inclusion criteria (Step 3). The search terms applied to full texts of publications in the PubMed database were as follows: “economic evaluation” + “country” or “cost” + “country”. The extended search for key words within abstracts (“economic evaluation” + “country”, “cost*” +”country”) was conducted in the Science Direct and Scopus databases (Figure 1). The difference in search conditions among databases was highlighted by the unlimited number of word combinations appearing if the term “cost*” was searched in PubMed.

The following exclusion criteria were applied for Step 1: study older than 5 years (<2008) based on the publication-date of the ISPOR task force report on transferability in 2009 (10); abstract not available, study written in a language other than English. The inclusion criteria for Step 1 were the following: study includes at least one country of interest, study includes an economic evaluation, study published as a peer-reviewed article (abstract only, or congress report excluded) in the English language.

Full texts of the publications were analyzed on correspondence to the inclusion criteria during the second step: study includes comparative economic evaluation, conducted in healthcare setting of at least one country of interest, full text of the study available. Both trial-based and model-based economic evaluations were eligible for inclusion. All references included in the second step were summarized in the dataset with the following information: main author and year of publication, whether it is an economic evaluation or not, inclusion of a direct comparison of two or more technologies, countries included in the assessment, full text availability, decision on inclusion in the systematic review. Hard copies of potentially relevant full-text articles were received. Authors of the articles which corresponded to all inclusion criteria except full-text availability were contacted. If no full text of the article was received, the publication was withdrawn from the analysis, and the reason for this was recorded.

### Data Extraction and Reporting

The following information was summarized from the included studies: (i) Technical characteristics of the publications: country and affiliation of the main and corresponding author (if differ), study sponsorship and type of sponsorship indicated. (ii) Study characteristics: countries of analysis, clinical area, study technology and comparators, type of analysis and methods used, perspective, application of discounting, costs assessment (technology, medical, and productivity costs), outcome measure, type of sensitivity analysis applied. (iii) Quality assessment using Drummond's check-list for assessing economic evaluations (11). (iv) Use of local inputs for the main data categories according to Barbieri et al. (12): baseline risk, treatment effect, health state preference values (utilities), resource usage, unit costs (prices). (v) Addressed limitations regarding foreign data use and transferability of the received results to other jurisdictions.

The articles were assessed independently by two researchers (O.M. and either S.K., Z.K., or J.L.S.). The results of the two independent assessments during the third step were compared, and any disagreements were discussed. If no consensus was reached, a third researcher was involved in the final decision making. The authors will provide their assessments for each of the included article on request.

## RESULTS

Of the forty-seven full-text publications, thirty-four articles (13–46) were included in the systematic review. Fifteen of the studies (44 percent) had a main author (and corresponding authors, if different) not from a study country (Western European countries, the United Kingdom, or the United States). An academic affiliation was the most common affiliation of the main author (25 or 74 percent of the studies). The study's sponsorship was indicated in twenty-two (65 percent) publications; of these, pharmaceutical companies sponsored five and conducted two more studies.

### Background Characteristics of Economic Evaluations

The main characteristics of the studies are described in Table 1. The majority of the retrieved studies were conducted in the healthcare settings of Bulgaria, the Russian Federation, Slovenia, Lithuania, and Ukraine. The retrieved studies also included six cross-country studies, which additionally analyzed the application of a technology in Croatia, Tajikistan, and Uzbekistan. Infectious diseases were the most frequently addressed topics in publications (16 or 47 percent of all studies), and the most frequently funded (87 percent of infectious disease studies were funded in comparison to 44 percent of all the other evaluations). Besides pharmaceutical companies, the other sponsors of studies on infectious diseases were international organizations, European and the U.S. grant committees, Ministries of Health or universities. In studies considering chronic diseases, different cardiologic interventions and diabetes medicines were the most frequently addressed. Medicines were the most frequently researched interventions, among which vaccines had a significant share. Healthcare, governmental, or healthcare payer perspectives were predominant in the analyzed publications. Models were applied in two-third of the studies (Markov model was frequently used). Cost-utility analysis, with quality-adjusted life-years (QALY) at the effect side, was applied in more than half of the evaluations.

**Table 1.** Characteristics of the Included Economic Evaluations

Parameters	N (%)^a,b^
Perspective of the study is stated^c^	23 (68%)
Health care, state, or health care payer	25 (74%)
Societal	5 (15%)
Provider	3 (9%)
Employer	1 (3%)
Patient	1 (3%)
Discounting applied	21 (62%)
Both costs and outcomes are discounted at 3% (% from model studies)	10 (43%)
Both costs and outcomes are discounted at 5% (% from model studies)	4 (17%)
Unequal discounting for costs and effects (% from model studies)	4 (17%)
Productivity costs included outcomes used	5 (15%)
Quality adjusted life years^d^	18 (53%)
Life-years gained	13 (38%)
N of cases/deaths averted	6 (18%)
Disability adjusted life years	5 (15%)
Sensitivity assessed^e^	27 (79%)
Probabilistic sensitivity analysis reported (% from model studies)	11 (48%)
Only univariate analysis	11 (32%)
Only univariate with multivariate analyses	4 (12%)
Bootstrap	1 (3%)

^a^Thirty-four articles in total.

^b^Rounding is applied.

^c^Number of studies used several perspectives.

^d^One study assessed quality of life using WHOQOL-BREF instrument.

^e^Two studies indicated that sensitivity analysis was applied, but did not report the results.

### Quality of Economic Evaluations

The summary of the assessment of the articles are presented in Table 2. Using Drummond's check-list for assessing economic evaluations (including considerations of internal and external validity of the study, such as methodology applied and healthcare setting) it was observed that the following criteria were ranked as “no” and “unclear” in more than 30 percent of studies: perspective stated, all relevant costs included, costs measured accurately in appropriate units, outcomes and costs valued credibly, and uncertainty addressed. Appraisal criteria such as comprehensive description of alternatives given, all relevant outcomes included, outcomes measured accurately in appropriate units, outcomes and costs adjusted for different times, incremental analysis performed, and conclusions justified by the evidence presented, were ranked as “yes” more than other Drummond criteria. Insufficient information on costs components and assessment methods frequently made it impossible to evaluate the quality of these data.

**Table 2.** Quality and Transferability of the Included Economic Evaluations^a^

Parameters	Yes^b^	Partially^b^	No^b^	Unclear^b^
Comprehensive description of alternatives given	27 (79%)	0	6 (18%)	1 (3%)
Effectiveness is established	22 (65%)	3 (9%)	2 (6%)	7 (21%)
All relevant costs included	18 (53%)	0	6 (18%)	10 (29%)
All relevant outcomes included	29 (85%)	2 (6%)	3 (9%)	0
Costs measured accurately in appropriate units	17 (50%)	0	1 (3%)	16 (47%)
Outcomes measured accurately in appropriate units	26 (76%)	5 (15%)	1 (3%)	2 (6%)
Outcomes and costs valued credibly	13 (38%)	5 (15%)	3 (9%)	13 (38%)
Incremental analysis performed (33 applicable)^c^	27 (82%)	1 (3%)	4 (12%)	1 (3%)
Uncertainty addressed	12 (35%)	11 (32%)	9 (26%)	2 (6%)
Results include issues of purchasers concern	18 (53%)	7 (21%)	5 (15%)	4 (12%)
Conclusions justified by the evidence presented	25 (74%)	4 (12%)	5 (15%)	0
Results can be applied to the local population	31 (91%)	1 (3%)	1 (3)%	1 (3%)
Unit costs retrieved from local data	28 (82%)	4 (12%)	1 (3%)	1 (3%)
Resource utilization retrieved from local data	23 (68%)	2 (6%)	1 (3%)	8 (24%)
Utility parameters retrieved from local data (19 applicable)^c^	1 (5%)	0	18 (95%)	0
Baseline risk received from local data (32 applicable)^c^	23 (72%)	2 (6%)	7 (22%)	0
Treatment effect received from local data	15 (44%)	2 (6%)	16 (47%)	1 (3%)
Transferability of study to other jurisdiction was discussed	4 (12%)	8 (24%)	22 (65%)	–
Limitations of the results regarding foreign data used (25 applicable)^c^	4 (16%)	4 (16%)^a^	17 (68%)	–

^a^Thirty-four articles in total.

^b^Rounding is applied.

^c^Percentage is indicated from applicable.

### Address of Transferability in Economic Evaluations

In ten of the twenty-three studies, the use of a country-adapted model was clearly stated. The frequencies on other transferability issues ranking are presented in Table 2. Unit costs were most frequently based on local data; there was only one study which did not apply local unit costs. The baseline risk and resource usage were also frequently assessed using local inputs, while the utility parameters were clearly identified as local in only one study.

Limitations of the results regarding the use of foreign data were discussed at least partially in eight studies, and twelve studies at least partially discussed the transferability of study results to other jurisdictions. Several studies briefly discussed the generalizability of the results or individual parameters (such as baseline risk, prevalence), while the studies of Berkhof et al. (15) and Winetsky et al. (46) generalized the received results to the other countries of the region (or conducted a simple transferability assessment).

## DISCUSSION

The results of this systematic review of economic evaluations conducted in CEE and former Soviet countries allowed us to conclude a low transparency of data reporting in the analyzed publications as well as insufficient consideration of inputs and results transferability in these studies.

### Background Characteristics of Economic Evaluations

We did not observe any proportional difference in the number of available English-language peer-review publications referenced in the international databases based on the country's size or level of HTA development. While the topic of the study may be sponsorship-driven, the number of publications submitted to international journals may depend on the publication activity of the local research teams. This conclusion is supported by the observation that, in the countries with a relatively high number of publications (such as Bulgaria and Lithuania), the articles are frequently published by the same teams. Additionally, we observed a trend for sponsored studies and for studies conducted under international co-authorship (either the first author or the corresponding author is not affiliated with the study country) to be of higher quality as assessed by Drummond's criteria. While it could be noticed that studies on some technologies were of better quality (e.g., vaccines), we believe that the main factor influencing study quality is the authors’ affiliation and source of sponsorship. A similar observation of higher quality of economic studies conducted by international rather than local teams was made in a systematic review of economic studies in Vietnam (47).

While it appears that medical interventions other than pharmaceuticals as well as studies on chronic conditions may be of higher interest for the decision makers, the analyzed publications tend to present more analyses related to drug treatment, especially vaccination, and focus more on infectious diseases than on chronic diseases.

Despite the fact that most guidelines on economic evaluations recommend using the societal perspective (48), its application in CEE and former Soviet countries is limited. Only a few studies used a limited or not purely societal perspective as defined by the ISPOR task force report (49). Data availability and decision-makers’ acceptance are the key factors in defining the perspective of the study (10) which, in the studied countries, majorly concern healthcare or third-party payers.

### Quality of Economic Evaluations

Insufficient quality of economic evaluations is the first knock-out criterion in assessment of studies’ transferability (50) and lack of transparency in the reporting of economic evaluations is the major concern of decision makers around the world (3;51). At the same time, we observed a significant indistinctness in reporting the methodology of economic evaluations conducted in healthcare settings of CEE and former Soviet countries. This reporting approach may improve by using standardized instruments, such as the CHEERS statement (52).

Absence of a clearly stated perspective of the study causes difficulties in the assessment of both the credibility of the study and its application in the decision making context. The description of the economic model used and its authorship was frequently lacking. Together with missing reporting on internal and external (between-model) validation (53), this may create difficulties for the transferability of study results.

While costs choice fully depends on the perspective of the analysis, their values and measurements should be transparent, appropriately documented and available for readers (49). However, incompleteness of data, the sample size required to estimate population-representative costs and effects, data heterogeneity, and generalizability of trials’ results are required (53), but rarely reported, in the trial-based economic evaluations conducted in healthcare settings of CEE and former Soviet countries. At the same time in countries with high data uncertainty, comprehensive probabilistic sensitivity assessment in modeled studies may improve the perceived quality (or reliability) of a study and thus the use of economic evaluations in the decision-making process (3).

### Consideration of Transferability of Economic Evaluations

While economic evaluations conducted in CEE and former Soviet countries typically apply local costs, baseline risk and resource usage measurement, the effectiveness and utilities are frequently extrapolated from other countries or multinational studies. This observation corresponds to the conclusions of other authors defining baseline risk, unit costs, and resource use as parameters of low transferability (12;54).

Many guidelines recommend using utility values from the jurisdiction of interest (10). The evidence suggests that utilities may vary between countries (55). Meanwhile, taking into account the data constraints, the decision makers from CEE and former Soviet countries may review generalizability of outcomes while addressing its uncertainty using statistical approaches.

Moreover, we observed that the limitations of foreign data use, as well as the possibility of transferring the study to other jurisdictions, are rarely described in the analyzed publications. Clear presentation of these parameters together with defining major impact factors on the results of economic evaluations and addressing data uncertainty will improve the transferability of studies.

### Policy and Future Research Considerations

Because of the limited HTA capacity, geographic transferability is an important alternative to conducting country-specific economic studies (56). Meanwhile, CEE and former Soviet countries require an adapted approach to addressing the use and transferability of economic evaluations in healthcare decision making. Because of the information (data and knowledge) constraints, this approach may not always correspond to the international guidelines on economic evaluations or practices used in HTA-experienced countries. As such, healthcare or third-party payer perspectives may be preferable to a societal one, and the generalizability of utilities may be considered to be appropriate, while local data should be used for baseline risk, unit costs, and resources consumption. The decision-makers’ preferences in these countries should be analyzed to understand the importance and relevance of studies’ methodology and possible impact of economic evaluations on a decision-making process.

### Limitations

This study is limited due to the use of the following study selection criteria: (i) English-language publications only, (ii) studies published from 2008 onward, (iii) articles with full-text availability. Search limitations could result in noninclusion of some relevant studies. Drummond's criteria were used to assess the quality of the economic evaluations. This instrument is a general questionnaire and does not provide a total scoring of the quality of the assessed papers, leaving the conclusion on each article to the subjective judgment of the people assessing it. The limited number of selected articles causes that the study does not have the statistical power to provide an assessment of relationships between different characteristics.

## CONCLUSION

Transferability of economic evaluations, conducted in healthcare settings of CEE and former Soviet countries is limited by a low number of English language peer-reviewed studies especially in chronic diseases, underreporting of methodology in publications, and limited discussion on inputs and results transferability. To improve the transferability of published studies to other jurisdictions, uncertainty, the impact of influential parameters, and data transferability should be comprehensively addressed when reporting studies. Additionally, the transparency of study reporting, especially study perspective, model details, and costing methodology, should be improved significantly.

## CONFLICTS OF INTEREST

The authors have no conflict of interest to declare.
